# Molecular genetics analysis of hereditary breast and ovarian cancer patients in India

**DOI:** 10.1186/1897-4287-7-13

**Published:** 2009-08-06

**Authors:** Nagasamy Soumittra, Balaiah Meenakumari, Tithi Parija, Veluswami Sridevi, Karunakaran N Nancy, Rajaraman Swaminathan, Kamalalayam R Rajalekshmy, Urmila Majhi, Thangarajan Rajkumar

**Affiliations:** 1 Department of Molecular Oncology, Cancer Institute (WIA), Chennai, India; 2 Currently: Department of Genetics, Sankara Nethralaya, Chennai, India; 3 Department of Surgical Oncology, Cancer Institute (WIA), Chennai, India; 4 Department of Epidemiology and Tumour Registry, Cancer Institute (WIA), Chennai, India; 5 Department of Hematology and Immunology, Cancer Institute (WIA), Chennai, India; 6 Department of Pathology, Cancer Institute (WIA), Chennai, India

## Abstract

**Background:**

Hereditary cancers account for 5–10% of cancers. In this study *BRCA1*, *BRCA2 *and CHEK2*(1100delC) were analyzed for mutations in 91 HBOC/HBC/HOC families and early onset breast and early onset ovarian cancer cases.

**Methods:**

PCR-DHPLC was used for mutation screening followed by DNA sequencing for identification and confirmation of mutations. Kaplan-Meier survival probabilities were computed for five-year survival data on Breast and Ovarian cancer cases separately, and differences were tested using the Log-rank test.

**Results:**

Fifteen (16%) pathogenic mutations (12 in *BRCA1 *and 3 in *BRCA2*), of which six were novel *BRCA1 *mutations were identified. None of the cases showed CHEK2*1100delC mutation. Many reported polymorphisms in the exonic and intronic regions of *BRCA1 *and *BRCA2 *were also seen. The mutation status and the polymorphisms were analyzed for association with the clinico-pathological features like age, stage, grade, histology, disease status, survival (overall and disease free) and with prognostic molecular markers (ER, PR, c-erbB2 and p53).

**Conclusion:**

The stage of the disease at diagnosis was the only statistically significant (p < 0.0035) prognostic parameter. The mutation frequency and the polymorphisms were similar to reports on other ethnic populations. The lack of association between the clinico-pathological variables, mutation status and the disease status is likely to be due to the small numbers.

## Introduction

Breast cancer is the most common cancer among women in Chennai with the crude incidence rate (CIR) being 30.1/100,000 in the Madras Metropolitan Tumor Registry (MMTR). A significant increasing trend in the incidence of breast cancer was seen during 1982–2005 with an average annual increase of 0.72 per 100,000 [1]. The trend of rising incidence rate is likely to continue due to further changes in life style factors such as age of first child birth and dietary habits [2].

*BRCA1 *and *BRCA2 *genes had been identified by linkage analysis and positional cloning on large breast cancer families in the early 1990's [3,4]. This has improved the understanding of the molecular genetics of the hereditary breast and ovarian cancer thus providing scope for better management in these patients and in offering predictive testing, aggressive screening and preventive strategies to the unaffected carriers in the same family. The CHEK2*1100delC mutation confers a low risk of breast cancer in non-*BRCA1*/*BRCA2 *mutation carriers [5]. The average cumulative risk of breast cancer in a *BRCA1 *and *BRCA2 *mutation carrier is 65% and 45% by the age of 70 years, respectively. For ovarian cancer the risk is 39% and 11%, in *BRCA1 *and *BRCA2 *mutation carriers by 70 years, respectively [6]. Slightly elevated risk of colon and prostate cancer in *BRCA1 *mutation carriers and increased risk of other malignancies including pancreatic, stomach and head and neck cancers and a large increase in the relative risk for male breast cancer is seen in *BRCA2 *mutation carriers [7,8].

Other genes like *TP53, ATM, SKT11, PTEN, PTCH MLH1, MSH2, PMS1, PMS2, MSH6 *have been associated with increased risk of breast and ovarian cancer as part of other cancer syndromes [9]. Recently genome wide association study identified four plausible causative genes namely *FGFR2, TNRC9, MAP3K1 *and *LSP1 *that confer moderate susceptibility to breast cancer [10].

Generally, hereditary breast cancers are earlier in onset and have a higher prevalence of bilateral breast cancers. Breast tumors from *BRCA1*- carriers are more likely to be highly proliferative; poorly differentiated (grade III); more likely to have a medullary or atypical medullary-like appearance with high degree of lymphocyte infiltration; and show excess of continuous pushing margins [11]. Moreover these tumors are more often ER, PR and c-erbB2 negative and frequently show p53 alterations [12]. Lakhani and Bell et al report *BRCA2 *tumors to be of higher grade than the sporadic group, but this was not confirmed by Eelora et al, 2005 [13-15]. The *BRCA2 *tumors are likely to be ER positive [16]. *BRCA1 *mutation carriers have an earlier onset of ovarian cancer that is histologically high-grade serous adenocarcinoma [17]. We had earlier published the data of *BRCA1*, *BRCA2 *and CHEK2*1100delC mutation status in 22 cases [18]. Here, we report the clinico-pathological features, *BRCA1*, *BRCA2 *and CHEK2*1100delC mutation status and their correlation with prognostic markers in 91 cases.

## Materials and methods

### Materials

Ninety-one eligible cases including the 22 published that fulfilled the criteria for gene testing were screened for *BRCA1*, *BRCA2 *and CHEK2*1100delC mutations [18]. The criteria were as follows: Early onset of breast cancer (≤35 years of age) or ovarian cancer (≤40 years of age); Two cases of breast cancer diagnosed under the age of 50 years in a family (first and second degree relatives); Three or more cases of breast cancer diagnosed at any age; Presence of breast and ovarian cancer in the family or in the same individual; Male breast cancer with a relative (of either sex) with breast cancer; Family history of Prostate or pancreatic or colorectal cancer or sarcomas, with breast cancer in the family. The study includes 65 unilateral, 6 bilateral breast cancer cases, 14 ovarian cancer cases, 3 cases with both breast and ovarian cancer in the same individual without any family history of cancer, 2 cases each with family history of breast cancer and soft tissue sarcoma and 1 case of prostate cancer with family history of prostate and breast cancer. Of the 91 cases, 54 were with Family history (including 35 Hereditary Breast Cancer families, 15 Hereditary Breast & Ovarian Cancer families, 3 Hereditary Ovarian Cancer families and 1 with family history of Prostate and breast cancer). There were 31 early onset breast cancer (≤ 35 yrs) and 3 early onset ovarian cancer (≤ 40 yrs) cases without family history of cancer. Three patients had breast and ovarian cancers, without a family history. All the patients provided their informed consent for the study, which was cleared by the Institutional Ethical committee.

### Methods

#### Mutation screening of *BRCA1*, *BRCA2 *and CHEK2*1100delC

The sample collection, DNA isolation, PCR-DHPLC and sequencing were carried out as previously described [18].

#### Bioinformatics Analysis

The secondary structure and the tertiary structure of the normal *CHEK2 *gene sequence and the two missense variants were predicted by submitting the sequences to Chou Fasman secondary structure prediction site and at ExPaSy server for tertiary structure prediction using Swiss model [19-22].

#### Immunohistochemistry

IHC was done for ER, PR, p53 and c-erbB2 expression studies on available breast cancer cases. IHC was performed as per the standard methodology using primary antibodies; clone ID5 (ER), clone PgR636 (PR), clone HER2/neu (c-erbB2), and clone DO7 (p53) and secondary antibodies; rabbit anti mouse for ER, PR and p53 and swine anti rabbit for c-erbB2 from Dako [23-26]. The antibodies were used at a dilution of 1:35 for ER and PR (Dako pharma), 1:350 for HER2/neu (Dako pharma) and 1:200 (Biogenex) or 1:50 (Dako pharma) for p53. Positive (known positives for each of the markers) and negative controls (omission of primary antibody) were included in each run. The scoring was done as described previously [23-26].

### Statistical Analysis

Chi-square test of independence was used for testing the statistical significance of association between the variables like age, stage, grade and histological type with disease status (i.e. disease free or disease present) and between clinico-pathological features and with each of the prognostic markers like ER, PR, c-erbB2 and p53 status for the breast cancer cases. The *BRCA1 *and *BRCA2 *mutation status i.e. mutation positivity or negativity were tested for statistical association with disease status and prognostic molecular markers. Yates correction was made wherever indicated. Fisher's exact probability test was employed to deal with small frequencies. Kaplan-Meier survival probabilities were computed for five-year survival data on Breast and Ovarian cancer cases separately, and differences were tested using the Log-rank test. All the statistical analyses were performed using Stata version 10.0 software.

## Results

### *BRCA1 *and *BRCA2 *analysis

Fifteen out of the 91 (16%) samples analyzed had a deleterious mutation. Twelve mutations were in *BRCA1 *and three in *BRCA2 *gene. The mutations and genetic variants were designated according to HUGO recommendations. The gene, mutation, exon, and the details of age of onset and family history are given in Table 1. Six of the 15 pathogenic mutations were novel and have been submitted to the GenBank .

**Table 1:** Gene, exon, mutation description, site of cancer in proband with age of onset and family history

**S. No**	**Gene**	**Exon**	**Mutation**	**Site of Cancer**	**Age of onset**	**Family History**
1	BRCA1	12	c.4158_4162delCTCTC; p.Ser1369SerfsX2 [**Acc No**. AY144588]*	Breast	28	Mother – B (44 y) MA – B (50 y) MGM – B (50 y)

2	BRCA1	13	c.4327C>T; p.R1443X *	Breast	40	3 Sisters – B (35 y, 38 y, 48 y) PA – B (64 y)

3	BRCA1	11C	c.1148_1149delAT; p.Asn383Arg fsX6 **[Acc No**. AY458144]	Breast	38	2 Sisters (50 and 49 y)

4	BRCA1	14	c.4399C>T; p.Gln1467X [**Acc No**. AY06912]	Breast (Metachronous Bil.) and uterine	38 (L) 40 (R) 43 (En)	2 sisters – B (39 y) Mother – O (74 y)

5	BRCA1	16	c.4705_4706insTGGAATC; p.Ile1567fsx5 [**Acc No**. DQ075361]	Breast	45	Mother – O (58 y)

6	BRCA1	17	c.5024_5025insT; p. Thr1675Thr fsX4 [**Acc No**. AY706913]	Ovarian	61	Daughter – O (39 y)

7	BRCA1	2	c.68_69delAG; p.Glu23Val fsX16	Breast	33	No family history

8	BRCA1	2	c.68_69delAG; p.Glu23Val fsX16	Breast	33	No family history

9	BRCA1	2	c.68_69delAG; p.Glu23Val fsX16	Breast	39	Mother – O (60 y)

10	BRCA1	2	c.68_69delAG; p.Glu23Val fsX16	Ovarian	59	Sister – O (44 y) Daughter – B (39 y)

11	BRCA2	11O	c.6214_6218delCTTAA; p.Ser2072Ser fsX4 *	Breast	38	Mother – B (40 y)

12	BRCA2	1D	c.5130_5133delTGTA; p.Tyr1693X	Breast	27	No family history

13	BRCA1	2	c.66_67delAG; p. Leu22Leu fsX18	Breast	55	Mother – B (41 y) O (55 y) MA – B (47 y)

14	BRCA1	18	c.5118_5120delAAT; p.del1707Ile [**Acc No**. FJ940752]	Breast	45	Mother – B (39 y) MA – B (64 y) Sister – B (33 y)

15	BRCA2	11G	c.2621_2627delAACTGTC; p. Ile873Ile fsX19	Breast	35	Mother – Cervix (40 y) MGM – B (60 y) PA – U (52 y)

B-Breast cancer, O-ovarian cancer, MA-Maternal aunt, PA-Paternal aunt, MGM-Maternal grandmother, Acc No-GenBank accession number, * – Mutations already reported in our previous publication [18]

Apart from the pathogenic mutations, many known missense variations both in the exonic and the intronic regions of *BRCA1 *and *BRCA2 *genes were seen in our study. The common missense variations in the exons and introns of *BRCA1 *and *BRCA2 *gene are given in Table 2. 85/91 (93%) cases showed at least one variation either in the exon or intron of *BRCA1 *or *BRCA2 *gene. Six cases did not show any variations either in exon or intron of both *BRCA1 *and *BRCA2 *gene. 28/91 (31%) did not show any variations in *BRCA1 *and 20/91 (22%) cases did not show any variations in *BRCA2 *gene.

**Table 2:** The missense variations in the exons and the introns (defined polymorphisms in BIC database) of *BRCA1 *and *BRCA2 *gene and their percentage

	**BRCA1**		**BRCA2**	
	
	**Polymorphisms**	**Cases (n = 91)** **(%)**	**Polymorphisms**	**Cases (n = 91)** **(%)**
**Exons**			3 – c.294A>G; p. L98L *	1 (1%)
	11K – c.2311T>C; p. L771L	42 (45.4%)	10B – c.1114C>A; p. H372N	36 (40%)
	11N – c.2612C>T; p. P871L	38 (41.2%)	10C – c.1365A>G; p. S455S	1 (1%)
	11S – c.3113A>G; p. E1038G	20 (22%)	11B – c.4258G>T; p. D1420Y	2 (2%)
	11TU – c.3548A>G; p.K1183R	12 (13%)	11C – c.4779A>C; p. E1593D	3 (3.1%)
	16 – c.4956G>A; p. M1652I	4 (4.1%)	11F – c.2538A>C; p. S846S *	2 (2%)
	16 – c.4838A>G; p. S1613G	4 (4.1%)	11H – c.2892A>T; p. K964N *	1 (1%)
	11P – c.2597G>T; p. R866L *	1 (1%)	11I – c.2971A>G; p. N991D	5 (5.3%)
			11K – c.3807T>C; p. V1269V	4 (4.2%)

**Introns**	c.301-41T>C	5 (5.3%)		
	c.441-34T>C	1 (1%0	c.1-26G>A	
	c.548-58del T	22 (24%)	c.6841+79delTTAA	4 (4.2%)
	c.4184-10G>A	1 (1%)	c.7807-14T>C	33 (36%)
	c.4987-68A>G	22 (24%)	c.8755-66T>C	39 (43.2%)
	c.4987-92A>G	22 (24%)	c.68-21T>G	38 (42%)
	c.5075+66G>A	5 (5.3%)	c.7804-12T>C	1 (1%)
	c.5152+66A>G	2 (2%)		1 (1%)
	c.547-64 del T	1 (1%)		

### CHEK2*1100delC mutation analysis

**Figure 1:**
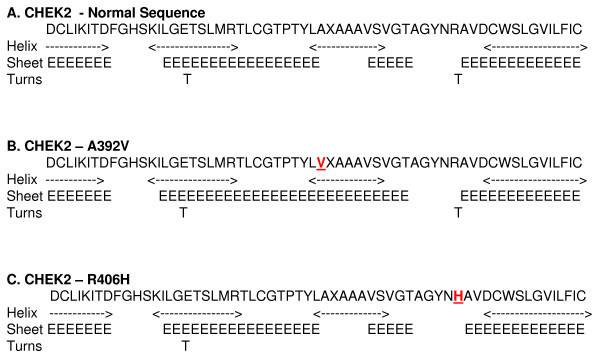
**Secondary structure as predicted by Chou – Fasman algorithm**. a) Normal sequence. b) CHEK2 A392V. c) CHEK2 R406H. The missense variations valine and histidine are typed in bold and underlined.

**Figure 2:**
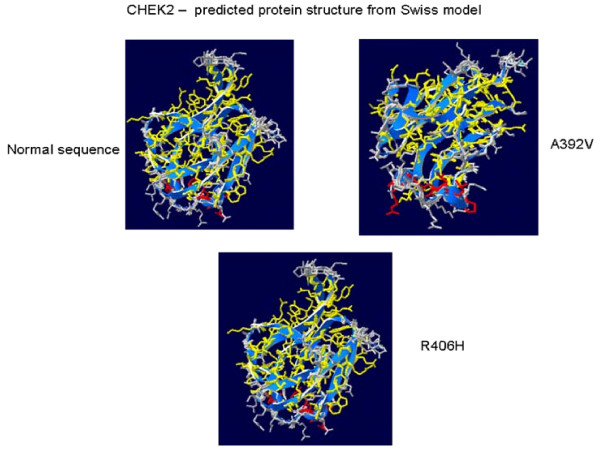
**Swiss model predicted protein structures of normal, A392V and R406H CHEK2 sequences**.

In our study none of the 91 samples analyzed showed the CHEK2*1100delC mutation. However, two missense mutations were seen in three cases. c.1217G>A; p.R406H which is a known missense mutation was seen in two patients, both with breast cancer and a family history of breast cancer. The second missense variation was c.1175C>T;p.A392V seen in an early onset breast cancer case without any family history of cancer. The Chou Fasman secondary structure prediction showed extension of β sheet and loss of a turn in the A392V and R406H missense mutation, respectively when compared with the normal sequence (Figure 1). The Swiss model for tertiary structure prediction show marked variation in the structure of A392V missense change. The R406H missense mutation, however did not show any change in its tertiary structure in the Swiss model (Figure 2).

### IHC analysis, Clinico-Pathological Features and Statistical Analysis

In our study 10/15 *BRCA1 *mutation positive cases were breast cancer and 2/16 were ovarian cancer cases. 8/10 *BRCA1 *mutation positive breast cancer cases were ER and PR negative and 5/8 were p53 positive. c-erbB2 data was available for 6 cases, of which 5 were negative and 1 was positive. Of the three *BRCA2 *mutation positive cases data was available for only one case, which was negative for ER, PR and p53. Clinico-pathological variables like age, stage, grade, histology, ER, PR, c-erbB2 and p53 status for 65 unilateral, 6 bilateral breast cancers and 14 ovarian cancers are given in Table 3. Of the 91 cases, ER, PR, c-erbB2 and p53 data was available only for 59, 57, 50 and 48 cases respectively. Data was not available for 32 cases; 10/32 patients were being managed outside the Institute and either declined or were unable to provide paraffin blocks. In the remaining cases only FNAC was done for the diagnosis of the disease, and not enough slides were available for all the marker study.

**Table 3:** Clinico-pathological characteristics of breast and Ovarian cancer cases

**Clinico-pathological features**	**65 Unilateral breast cases in number (%)**	**6 Bilateral breast cases in number (%)**	**14 Ovarian cases in number (%)**
**Age (years)**			
35	37 (56.8%)	5 (83.3%)	6 (42.8%)
36–45	17 (26.1%)	1 (16.6%)	3 (21.4%)
46–55	9 (13.8%)	-	3 (21.4%)
56–65	1 (1.5%)	-	-
>65	1 (1.5%)	-	2 (14.2%)

**Stage**			
I	8 (12.5%)	Lt: 2(33.2%) Rt:1(16.6%)	2 (14.2%)
II	15 (23.0%)	Lt: 2(33.2%) Rt: -	-
III	37 (56.8%)	Lt: – Rt:4(66.6%)	7 (50%)
IV	2 (3.1%)	Lt: 2(33.2%) Rt:1(16.6%)	3 (21.4%)
NK	3 (4.6%)	-	2 (14.4%)

**Grade**			
I	30 (46.2%)	Lt:4(66.6%) Rt:1(16.6%)	3 (21.4%)
II	18 (27.3%)	Lt:1(16.6%) Rt:3(50%)	2 (14.2%)
III	10 (15.4%)	Lt: – Rt: -	3 (21.4%)
NK	7 (10.1%)	Lt:1 (16.6%)Rt:2(33.2%)	6 (42.8%)

**Disease Status**			
Disease free	43 (66.3%)	3 (50%)	5 (35.7%)
Recurrence & disease present	9 (14.0%)	1 (16.6%)	6 (42.8%)
Recurrence & disease free	1 (1.5%)	-	-
Default	6 (9.1%)	1 (16.6%)	1 (7.1%)
Not treated at CI	6 (9.1%)	1 (16.6%)	2 (14.2%)

**Type**			
IDC	53 (82.1%)	Lt:5(83.3%)Rt:5(83.3%)	-
Medullary	4 (6.2%)	-	
Mucinous	1 (1.6%)	-	
Others	3 (4.6%)	Lt:1(16.6%)Rt:1(16.6%)	
NK	4 (5.5%)	-	

**ER**			
Positive	15/55 (27.3%)	0	-
Negative	40/55 (72.7%)	4/4 (100%)	

**PR**			
Positive	17/53 (32.0%)	0	-
Negative	36/53 (68.0%)	4/4 (100%)	

**c-erbB2**			
Positive	15/46 (32.6%)	1/4(25%)	-
Negative	31/46 (67.4%)	3/4 (75%)	

**P53**			
Positive	21/44 (47.7%)	1/4(25%)	-
Negative	23/44 (52.3%)	3/4 (75%)	

Statistical analysis between clinico-pathological variables, disease status and prognostic markers revealed association between the stage of the disease at diagnosis and the disease status, which was statistically significant (p < 0.0035). There is a positive association between earlier stage at diagnosis and disease free status. No other variables like grade, age and histological types had significant association with the disease status and the prognostic molecular markers. In addition, no significant association between the mutation status and the clinico-pathological variables were seen.

**Figure 3:**
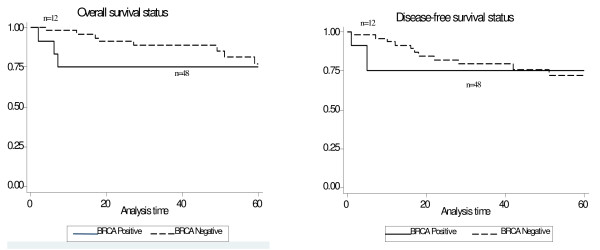
**Kaplan-Meier plot showing Overall survival and Disease free status for Breast cancer cases**.

**Figure 4:**
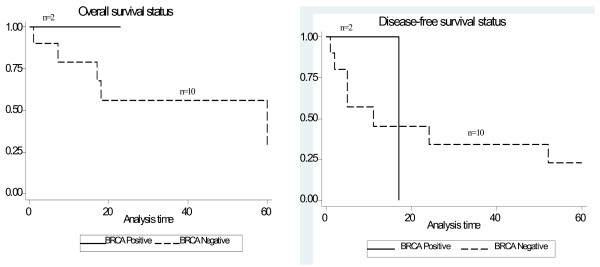
**Kaplan-Meier plot showing Overall survival and Disease free status for Ovarian cancer cases**.

The 5 year overall survival (OS) and disease-free survival (DFS) in breast cancer cases was 75% and 65% and for ovarian cancer cases was 30% and 22% respectively. The P_logrank _= 0.30 for overall survival and P_logrank _= 0.72 for disease-free survival in breast cancer cases and for ovarian cancer cases it is P_logrank _= 0.42 and P_logrank _= 0.89 respectively. The Kaplan-Meier curve for the breast and ovarian cancer cases are given in Figure 3 and Figure 4.

## Discussion

In our study on 91 patients, 15 pathogenic mutations (16%) in *BRCA1 *and *BRCA2 *genes were detected. This is similar to the data from several studies including the study by Wagner et al who had reported 17% mutation in either of the *BRCA *genes [27-31].

There is a four fold greater incidence of *BRCA1 *mutation (12) compared to BRCA2 (3) mutations in our series of South Indian patients. This is different from the North Indian data wherein almost equal distribution of *BRCA1 *and *BRCA2 *mutations was seen in two studies and in the study by Hedau et al [32,33], 4/24 mutations reported, were all in *BRCA1 *gene [34]. The two South Indian studies have looked at *BRCA1 *mutations only [35,36].

7/35 HBC families (20%) had mutations in either *BRCA1 *(5 mutations) *or BRCA2 *gene (2 mutations). Four of 15, HBOC cases were found to harbor mutations in *BRCA1 *gene (30%). Zelada-Hedman et al showed that 35% of the HBOC families have mutation in *BRCA1 *gene, but others report a higher percentage (58% and 43% respectively) of mutation in *BRCA1 *gene in HBOC families [28,37,38]. The German consortium (2002) study showed 10% mutation in *BRCA2 *gene in HBOC families [38]. In our series we did not find mutation in *BRCA2 *gene in HBOC families, which is similar to the results from a study in the Japanese population [39]. Three of 31 early onset (≤ 35 years) breast cancer cases had deleterious mutation, 2 in *BRCA1 *(7%) and 1 in *BRCA2 *(3%) gene. Peto et al also report 3.5% and 2.4% mutation in *BRCA1 *and *BRCA2 *gene, respectively in early onset (≤ 35 years) breast cancer cases [40]. Of the three hereditary ovarian cancer families studied, one had a deleterious mutation in the *BRCA1 *gene.

Results published from other Indian groups on *BRCA1 *&*BRCA2 *give varying results. Initial studies on smaller sample size gave higher percentage of mutation detected. Saxsena et al reported 2.9% (6/204) mutation with largest sample size so far analyzed [32]. However, this study includes 34 cases with family history and the rest being sporadic cases. The table 4 details the published results from the different groups from India [33-36,41]. The mutation c.68_69delAG; p.Glu23Val fsX16 was seen in four cases (two in HBOC families and two in early onset breast cancer case is the only recurrent mutation seen in this study. The above mentioned mutation has been identified as founder mutation in various populations like the Ashkenazi Jews; however haplotype analysis was not performed in these four families to check for common ancestor [28]. These four families are unrelated and are from different parts of South India, and ethnically different.

**Table 4:** Published results from various Indian groups on BRCA1 and BRCA2 mutation status on hereditary breast and ovarian cancer families or early onset breast cancer cases

**Total No. cases**	**Gene(s)**	**Mutation detected**	**Authors and Year**
14 cases with F/H of BC & OC	BRCA1	3/14 (21%)	Kumar et al (2002)

20 cases with F/H of B & OC or early onset (< 35 y)	*BRCA1 *&*BRCA2*	2/20 (10%) (*BRCA1*)	Saxsena et al 2002

24 cases with F/H of breast cancer and 100 sporadic cases	*BRCA*1 &*BRCA2*	6/24 (25%) (*BRCA1*)	Hedau el at 2004

16 cases F/H of BC & OC and 20 sporadic cases	*BRCA1 *&*BRCA2*	5/16 (31%) (*BRCA1 *&*BRCA2*)	Valarmathi et al 2004

23 cases with F/H of BC & OC	BRCA1	1/23	Gajalakshmi et al 2006

204 cases, 34 with F/H, 105 early onset (<40 y) and 65 late onset	*BRCA1 *&*BRCA2*	6/204 (2.9) (*BRCA1 *&*BRCA2*) except for one mutation were seen either in early onset cases or with F/H	Saxena et al 2006

91 cases, 54 with F/H of BC & OC, 34 early onset (≤ 35 y for BC and ≤ 40 y for OC); 3 HBOC in same individual	*BRCA1 *&*BRCA2*	15/91 (16%) (*BRCA1 *&*BRCA2*)	Current study

Many common missense variants were seen in our series both in the exon and intron of *BRCA1 *and *BRCA2*. Most have them been previously reported in other populations. The exonic variants seen in *BRCA1 *have been reported as unclassified variants in many studies [39,42,43]. In the BIC data base, most of the variants have been classified as having no clinical significance or of unknown clinical significance. Case control studies have shown no difference in the frequencies of these alleles in cases and controls [44]. However, we have not analyzed the frequency of the missense heterozygotes in our control population i.e. unaffected controls with out any family history of breast and/or ovarian cancer. The most common exonic missense variant of *BRCA2 *gene seen in our cases was N372H. Studies have shown that N372H appears to confer a small increased risk for breast cancer [44]. The missense variants seen in the intronic region of the *BRCA1 *gene and other less common exonic and intronic variants in *BRCA2 *have also been previously reported in other populations [28,37,38,40].

The CHEK2*1100delC mutation reported in the western populations were also screened for in our cases. Though no CHEK2*1100delC mutation was detected, two missense mutations in the *CHEK2 *gene (p.R406H and p.A392V) were seen in three cases. The residues, arginine and alanine at positions 406 and 392 are conserved across species. Additionally, the secondary structure prediction using the Chou-Fasman algorithm predicts changes in the secondary structure for both the missense variations [19].

The Swiss Model software too shows a marked change in the structure for the A392V missense variation, though the R406H did not show a visible change. The variation (R406H) was not seen to be associated with breast cancer cases in French Canadian women [45]. The pathogenicity of the variations can however be confirmed by segregation analysis in other affected family members or other functional studies or screening the normal controls. Two out of the three cases had a family history of breast cancer but we could not get the sample from the other affected family member for testing. The patient with the A392V mutation was an early onset breast cancer patient without any family history. Rest of the coding region of the *CHEK2 *gene was not screened for mutations.

In our series, nearly 80% of the breast cancer patients were ≤ 45 years of age at diagnosis, with 52.7% being ≤ 35 years at diagnosis, as in other studies [46]. Majority of the breast cancer cases were high-grade tumors (56%). Ho et al reported that 25%–37% of hereditary breast cancers not associated with *BRCA1 *mutations were ER negative but we found 68% to be negative for ER expression in our study [42]. This correlates well with our hospital statistics in sporadic cancers, wherein 62% of the pre-menopausal patients harbor tumours, which are ER negative.

Our study looked at the association between clinico-pathological features, disease status, and prognostic markers. We also looked at the association between the mutation status with disease status and prognostic markers. The stage of the disease at diagnosis and the disease status alone emerged to be statistically significant, wherein a positive association between earlier stage at diagnosis and disease free status was observed. None of the other parameters showed any association. Disease free and overall survival of *BRCA *mutation positive breast or ovarian cancers did not significantly differ from the *BRCA *mutation negative cases. Other groups have also not found any survival difference between *BRCA *mutation positive versus negative cases [46-49].

Of the 91 cases analyzed, 13 (14%) of the affected probands were issues of consanguineous marriage and had either ovarian cancers (3) or breast cancers (10). Though susceptibility to breast cancer is inhereited as an autosomal dominant trait, modifier genes when present in homozygous state might further increase the risk of cancer.

## Conclusion

We have looked at the prevalence of *BRCA1*, *BRCA2 *and *CHEK*1100delC mutation in breast and ovarain caner patients in India. The prevalence of *BRCA1 *and *BRCA2 *gene mutaion is similar to other population. *CHEK*1100delC mutation was not seen our study series. Asoociation between the clinico-pathological variables and mutation status with disease status and prognostic markers revealed significant assocaition between the stage at diagnosis and disease free status alone.

## Competing interests

The authors declare that they have no competing interests.

## Authors' contributions

SN, BM and TP carried out the sample collection, PCR-DHPLC, Sequencing, analysis of the data and drafting the article; VS was involved in sample collection, collection and analysis of all the clinical details and drafting of the manuscript; KNN was involved in data analysis and drafting of the article; RS was involved with all the statistical analysis of the data; KRR carried out the immunohistochemistry (IHC) and analysis of the IHC results; UM was involved in the pathological assessment of all the tumour samples; TR designed the study, was involved in the data analysis (clinical and lab), interpretation of the data, drafting the article. All the authors have read and approved the final version of the article.

## References

[B1] Shanta V, Swaminathan R (2008). Cancer incidence and mortality in Chennai, India 2003–2005. National Cancer registry program, Cancer Institute (WIA), Chennai.

[B2] Sue Moss (2008). Screening for breast cancer in India – is it an appropriate strategy?. J Natl Cancer Inst.

[B3] Miki Y, Swensen J, Donna Shattuck-Eidens, Futreal PA, Harshman K, Tavtigian S, Qingyun Liu, Cochran C, Bennett LM, Ding W, Bell R, Rosenthal J, Hussey C, Tran T, McClure M, Frye C, Hattier T, Phelps R, Haugen-Strano A, Katcher H, Yakumo K, Gholami Z, Shaffer D, Stone S, Bayer S, Wray C, Bogden R, Dayananth P, Ward J, Tonin P, Narod S, Bristow PK, Norris FH, Helvering L, Morrison P, Rosteck P, Lai M, Barrett JC, Lewis C, Neuhausen S, Cannon-Albright L, Goldgar D, Wiseman R, Kamb A, Skolnick MH (1994). A strong candidate for the breast and ovarian cancer susceptibility gene *BRCA1*. Science.

[B4] Wooster R, Bignell G, Lancaster J, Swift S, Seal S, Mangion J, Collins N, Gregory S, Gumbs C, Micklem G (1995). Identification of the breast cancer susceptibility gene *BRCA2*. Nature.

[B5] Heijboer HM, Ouweland Ans van den, Klijn J, Wasielewski M, Snoo A, Oldenburg R, Hollestelle A, Houben M, Crepin E, Veghel-Plandsoen M, Elstrodt F, Duijn C, Bartels C, Meijers C, Schutte M, McGuffog L, Thompson D, Easton DF, Sodha N, Seal S, Barfoot R, Mangion J, Chang-Claude J, Eccles D, Eeles R, Gareth Evans D, Houlston R, Murday V, Narod S, Peretz T, Julian P, Phelan C, Zhang HX, Szebo C, Derilee P, Goldgar D, Futreal PA, Nathanson KL, Weber BL, Rahman N, Stratton MR (2002). Low-penetrance susceptibility to breast cancer due to *CHEK2 **1100delC in non-carriers of *BRCA1*or *BRCA2 *mutations: The *CHEK2 *breast cancer consortium. Nat Genet.

[B6] Antoniou A, Pharoah PD, Narod S, Risch HA, Eyfjord JE, Hopper JL, Loman N, Olsson H, Johannsson O, Borg A, Pasini B, Radice P, Manoukian S, Eccles DM, Tang N, Olah E, Anton-Culver H, Warner E, Lubinski J, Gronwald J, Gorski B, Tulinius H, Thorlacius S, Eerola H, Nevanlinna H, Syrjäkoski K, Kallioniemi OP, Thompson D, Evans C, Peto J, Lalloo F, Evans DG, Easton DF (2003). Average risks of breast and ovarian cancer associated with BRCA1 or BRCA2 mutations detected in case Series unselected for family history: a combined analysis of 22 studies. Am J Hum Genet.

[B7] Ford D, Easton DF, Bishop DT, Narod SA, Goldgar DE (1994). Risks of cancer in *BRCA1*-mutation carriers. Breast Cancer Linkage Consortium. Lancet.

[B8] Robson ME (2002). Clinical Considerations in the Management of Individuals at Risk for Hereditary Breast and Ovarian Cancer. Cancer Control.

[B9] Marsh DJ, Zori RT (2002). Genetic insights into familial cancers-update and recent discoveries. Cancer Letters.

[B10] Easton DF, Pooley KA, Dunning AM, Pharoah PD, Thompson D, Ballinger DG, Struewing JP, Morrison J, Field H, Luben R, Wareham N, Ahmed S, Healey CS, Bowman R, Meyer KB, Haiman CA, Kolonel LK, Henderson BE, Le Marchand L, Brennan P, Sangrajrang S, Gaborieau V, Odefrey F, Shen CY, Wu PE, Wang HC, Eccles D, Evans DG, Peto J, Fletcher O, Johnson N, Seal S, Stratton MR, Rahman N, Chenevix-Trench G, Bojesen SE, Nordestgaard BG, Axelsson CK, Garcia-Closas M, Brinton L, Chanock S, Lissowska J, Peplonska B, Nevanlinna H, Fagerholm R, Eerola H, Kang D, Yoo KY, Noh DY, Ahn SH, Hunter DJ, Hankinson SE, Cox DG, Hall P, Wedren S, Liu J, Low YL, Bogdanova N, Schürmann P, Dörk T, Tollenaar RA, Jacobi CE, Devilee P, Klijn JG, Sigurdson AJ, Doody MM, Alexander BH, Zhang J, Cox A, Brock IW, MacPherson G, Reed MW, Couch FJ, Goode EL, Olson JE, Meijers-Heijboer H, Ouweland A van den, Uitterlinden A, Rivadeneira F, Milne RL, Ribas G, Gonzalez-Neira A, Benitez J, Hopper JL, McCredie M, Southey M, Giles GG, Schroen C, Justenhoven C, Brauch H, Hamann U, Ko YD, Spurdle AB, Beesley J, Chen X, Mannermaa A, Kosma VM, Kataja V, Hartikainen J, Day NE, Cox DR, Ponder BA, SEARCH collaborators, kConFab AOCS Management Group (2007). Genome-wide association study identifies novel breast cancer susceptibility loci. Nature.

[B11] Lakhani SR, Jacquemier J, Sloane JP, Gusterson BA, Anderson TJ, Vijver MJ, Farid LM, Venter D, Antoniou A, Storfer-Isser A, Smyth E, Steel CM, Haites N, Scott RJ, Goldgar D, Neuhausen S, Daly PA, Ormiston W, McManus R, Scherneck S, Ponder BA, Ford D, Peto J, Stoppa-Lyonnet D, Easton DF (1998). Multifactorial analysis of differences between sporadic breast cancer and cancers involving *BRCA1 *and *BRCA2 *mutation. J Natl Cancer Inst.

[B12] Berns EM, Van Staveren IL, Verhoog L, Van De Ouweland AM, Gelder MM, Meijers-Heijboer H, Portengen H, Foekens JA, Dorssers LC, Klijn JG (2001). Molecular profiles of *BRCA1*- mutated and matched sporadic breast tumors; relation with clinico-pathological features. British J Cancer.

[B13] Lakhani SR (1999). The pathology of familial breast cancer: morphological aspects. Breast Cancer Res.

[B14] Bell DW, Erban J, Sgroi DC, Haber DA (2002). Selective loss of heterozygosity in multiple breast cancers from a carrier of mutations in both *BRCA1 *and *BRCA2*. Cancer Res.

[B15] Eerola H, Heikkilä P, Tamminen A, Aittomäki K, Blomqvist C, Nevanlinna H (2005). A Histopathological features of breast tumours in *BRCA1, BRCA2 *and mutation-negative breast cancer families. Breast Cancer Res.

[B16] De Carvalho M, Jenkins J, Nehrebecky M, Lahl L (2003). The Role of Estrogens in *BRCA1/2 *Mutation Carriers. Cancer Nurs.

[B17] Rubin SC, Benjamin I, Behbakht K, Takahashi H, Morgan MA, LiVolsi VA, Berchuck A, Muto MG, Garber JE, Weber BL, Lynch HT, Boyd J (1996). Clinical And Pathological Features Of Ovarian Cancer In Women With Germ-Line Mutations Of *Brca1*. N Engl J Med.

[B18] Rajkumar T, Soumittra N, Nirmala KN, Swaminathan R, Sridevi V, Shanta V (2003). *BRCA1, BRCA2 And CHEK2 *(1100 Del C) Germline Mutations In Hereditary Breast And Ovarian Cancer Families In South India. APJCP.

[B19] FASTA/Hydropathy/Secondary-Structure/Seg. http://fasta.bioch.virginia.edu/fasta_www/chofas.htm.

[B20] Schwede T, Kopp J, Guex N, Peitsch MC (2003). SWISS-MODEL: an automated protein homology-modeling server. Nucleic Acids Research.

[B21] Guex N, Peitsch MC (1997). SWISS-MODEL and the Swiss-PdbViewer An environment for comparative protein modelling. Electrophoresis.

[B22] Peitsch MC (1995). Protein modeling by E-mail Bio/Technology.

[B23] Thorpe SM, Rose C (1986). Estrogen and progesterone receptor determinations in breast cancer technology and biology. Cancer Surv.

[B24] Lee A, Park WC, Yim HW, Lee MA, Park G, Lee KY (2007). Expression of c-ErbB2, Cyclin D1 and ER and their clinical implications in Invasive ductal carcinoma of breast. Jpn J Clin Oncol.

[B25] Gullick WJ (1989). Expression of c-ErbB2 protooncogene protein in human breast cancer. Recent results. Cancer Res.

[B26] Geisler S, Lønning PE, Aas T, Johnsen H, Fluge O, Haugen DF, Lillehaug JR, Akslen LR, Anne-Lise Børresen-Dale (2001). Influence of Tp53 gene alterations and c-erbB2 expression on the response to treatment with doxorubicin in locally advanced breast cancer. Cancer Res.

[B27] Wagner TM, Hirtenlehner K, Shen P, Moeslinger R, Muhr D, Fleischmann E, Concin H, Doeller W, Haid A, Lang AH, Mayer P, Petru E, Ropp E, Langbauer G, Kubista E, Scheiner O, Underhill P, Mountain J, Stierer M, Zielinski C, Oefner P (1999). Global sequence diversity of *BRCA2*: analysis of 71 breast cancer families and 95 control individuals of worldwide populations. Hum Mol Genet.

[B28] Wagner TM, Möslinger RA, Muhr D, Langbauer G, Hirtenlehner K, Concin H, Doeller W, Haid A, Lang AH, Mayer P, Ropp E, Kubista E, Amirimani B, Helbich T, Becherer A, Scheiner O, Breiteneder H, Borg A, Devilee P, Oefner P, Zielinski C (1998). *BRCA1 *– related breast cancer in Austrian breast and ovarian cancer families: specific *BRCA1 *mutations and pathological characteristics. Int J Cancer.

[B29] Risch HA, McLaughlin JR, Cole DE, Rosen B, Bradley L, Kwan E, Jack E, Vesprini DJ, Kuperstein G, Abrahamson JL, Fan I, Wong B, Narod SA (2001). Prevalence and Penetrance of Germline *BRCA1 *and *BRCA2 *Mutations in a Population Series of 649 Women with Ovarian Cancer. Am J Hum.

[B30] Palmieri G, Palomba G, Cossu A, Pisano M, Dedola MF, Sarobba MG, Farris A, Olmeo N, Contu A, Pasca A, Satta MP, Persico I, Carboni AA, Cossu-Rocca P, Contini M, Mangion J, Stratton MR, Tanda F (2002). *BRCA1 *and *BRCA2 *Germline mutations in Sardinian breast cancer families and their implications for genetic counseling. Ann Oncol.

[B31] Thirthagiri E, Lee SY, Kang P, Lee DS, Toh GT, Selamat S, Yoon SY, Taib NA, Thong MK, Yip CH, Teo SH (2008). Evaluation of *BRCA1 *and *BRCA2 *mutation and risk prediction models in Asian country (Malaysia) with a relatively low incidence of breast cancer. Breast Cancer Res.

[B32] Saxena S, Chakraborty A, Kaushal M, Kotwal S, Bhatanager D, Mohil RS, Chintamani C, Aggarwal AK, Sharma VK, Sharma PC, Lenoir G, Goldgar DE, Szabo CI (2006). Contribution of germline *BRCA1 *and *BRCA2 *sequence alterations to breast cancer in Northern India. BMC Medical Genetics.

[B33] Kumar BV, Lakhotia S, Ankathil R, Madhavan J, Jayaprakash PG, Nair MK, Somasundaram K (2002). Germline *BRCA1 *mutation analysis in Indian breast/ovarian cancer families. Cancer Biol Ther.

[B34] Saxena S, Szabo CI, Chopin S, Barjhoux L, Sinilnikova O, Lenoir G, Goldgar DE, Bhatanager D (2002). *BRCA1 *and *BRCA2 *in Indian breast cancer patients. Hum Mutat.

[B35] Hedau S, Jain N, Husain SA, Mandal AK, Ray G, Shahid M, Kant R, Gupta V, Shukla NK, Deo SS, Das BC (2004). Novel germline mutations in breast cancer susceptibility genes *BRCA *1, *BRCA*2 and p53 gene in breast cancer patients from India. Breast Cancer Res Treat.

[B36] Valarmathi MT, Sawhney M, Deo SS, Shukla NK, Das SN (2004). Novel germline mutations in the *BRCA1 *and BRCA2 genes in Indian breast and breast-ovarian cancer families. Hum Mutat.

[B37] Zelada-Hedman M, Wasteson Arver B, Claro A, Chen J, Werelius B, Kok H, Sandelin K, Håkansson S, Andersen TI, Borg A, Børresen Dale AL, Lindblom A (1997). A screening for *BRCA1 *mutations in breast and breast-ovarian cancer families from Stockholm region. Cancer Res.

[B38] Meindl A (2002). German consortium for hereditary breast and ovarian cancer: Comprehensive analysis of 989 patients with breast or ovarian cancer provides BRCA1 and BRCA2 mutation profiles and frequencies for the German population. Int J Cancer.

[B39] Ikeda N, Miyoshi Y, Yoneda K, Shiba E, Sekihara Y, Kinoshita M, Noguchi S (2001). Fequency of BRCA1 and BRCA2 germline mutations in Japanese breast cancer families. Int J Cancer.

[B40] Peto J, Collins N, Barfoot R, Seal S, Warren W, Rahman N, Easton DF, Evans C, Deacon J, Stratton MR (1999). Prevalance of *BRCA1 *and *BRCA2 *gene Mutations in patients with early-onset breast cancer. J Natl Cancer Inst.

[B41] Gajalakshmi P, Natarajan TG, Rani DS, Thangaraj K (2006). A novel *BRCA1 *mutation in an Indian family with hereditary breast/ovarian cancer. Breast Cancer Res Treat.

[B42] Ho GH, Phang BH, Ng IS, Law HY, Soo KC, Ng EH (2000). Novel germline *BRCA *1 mutations detected in women in Singapore who developed breast carcinoma before the age of 36 years. Cancer.

[B43] Friedman LS, Ostermeyer EA, Szabo CI, Dowd P, Lynch ED, Rowell SE, King MC (1994). Conformation of *BRCA *1 by analysis of germline mutations linked to breast and ovarian cancer in ten families. Nat Genet.

[B44] Nathanson KL, Weber BL (2001). "Other" breast caner susceptibility genes searching for more holy grail. Hum Mol Genet.

[B45] Novak DJ, Chen LQ, Ghadirian P, Hamel N, Zhang P, Rossiny V, Cardinal G, Robidoux A, Tonin PN, Rousseau F, Narod SA, Foulkes WD (2008). Identification of a novel *CHEK2 *variant and assessment of its contribution to the risk of breast cancer in French Canadian Women. BMC Cancer.

[B46] Verhoog LC, Brekelmans CT, Seynaeve C, Bosch LM van den, Dahmen G, van Geel AN, Tilanus-Linthorst MM, Bartels CC, Wagner A, Ouweland A van den, Devilee P, Meijers-Heijboer EJ, Klijn JG (1998). Survival and tumor characteristics of breast cancer patients with germline mutations of *BRCA1*. Lancet.

[B47] Johannsson OT, Ranstam J, Borg A, Olsson H (1998). Survival of *BRCA1 *breast and ovarian cancer patients: a population-based study from southern Swden. J Clin Oncol.

[B48] Lee JS, Wacholder S, Struewing JP, McAdams M, Pee D, Brody LC, Tucker MA, Hartge P (1999). Survival after breast cancer in Ashkenazi Jewish *BRCA1 *and *BRCA2 *mutation carriers. J Natl Cancer Inst.

[B49] Robson M, Gilewski T, Haas B, Levin D, Borgen P, Rajan P, Hirschaut Y, Pressman P, Rosen PP, Lesser ML, Norton L, Offit K (1998). *BRCA*-aasociated breast cancer in young women. J Clin Oncol.

